# Within-host modeling of primaquine-induced hemolysis in hemizygote glucose-6-phosphate dehydrogenase deficient healthy volunteers

**DOI:** 10.1128/aac.01549-24

**Published:** 2025-02-24

**Authors:** James A. Watson, Parinaz Mehdipour, Robert Moss, Podjanee Jittamala, Sophie Zaloumis, David J. Price, Saber Dini, Borimas Hanboonkunupakarn, Pawanrat Leungsinsiri, Kittiyod Poovorawan, Kesinee Chotivanich, Germana Bancone, Robert J. Commons, Nicholas P. J. Day, Sasithon Pukrittayakamee, Walter R. J. Taylor, Nicholas J. White, Julie A. Simpson

**Affiliations:** 1 Infectious Diseases Data Observatory, Oxford, United Kingdom; 2 Centre for Tropical Medicine and Global Health, Nuffield Department of Medicine, University of Oxford 105596 , Oxford, United Kingdom; 3 Centre for Epidemiology and Biostatistics, Melbourne School of Population and Global Health, University of Melbourne 559658 , Melbourne, Victoria, Australia; 4 Mahidol Oxford Tropical Medicine Research Unit, Faculty of Tropical Medicine, Mahidol University 115374 , Bangkok, Thailand; 5 Department of Infectious Diseases, Peter Doherty Institute for Infection and Immunity, University of Melbourne 534133 , Melbourne, Victoria, Australia; 6 Medical Therapeutics Unit, Faculty of Tropical Medicine, Mahidol University 115374 , Bangkok, Thailand; 7 Shoklo Malaria Research Unit, Mahidol-Oxford Tropical Medicine Research Unit, Faculty of Tropical Medicine, Mahidol University 26685 , Mae Sot, Thailand; 8 Global Health Division, Menzies School of Health Research and Charles Darwin University 2306 , Darwin, Northern Territory, Australia; 9 General and Subspecialty Medicine, Grampians Health-Ballarat 72558 , Ballarat, Victoria, Australia; The Children's Hospital of Philadelphia, Philadelphia, Pennsylvania, USA

**Keywords:** primaquine, G6PD deficiency, hemolysis

## Abstract

Primaquine is the only widely available drug to prevent relapses of *Plasmodium vivax* malaria. Primaquine is underused because of concerns over oxidant hemolysis in glucose-6-phosphate dehydrogenase (G6PD) deficiency. A pharmacometric trial showed that ascending-dose radical cure primaquine regimens causing ‘slow burn’ hemolysis were safe in G6PD-deficient Thai and Burmese male volunteers. We developed and calibrated a within-host model of primaquine hemolysis in G6PD deficiency, using detailed serial hemoglobin and reticulocyte count data from 23 hemizygote deficient volunteers given ascending-dose primaquine (1,523 individual measurements over 656 unique time points). We estimate that primaquine doses of ~0.75 mg base/kg reduce the circulating lifespan of deficient erythrocytes by ~30 days in individuals with common Southeast Asian *G6PD* variants. We predict that 5 mg/kg primaquine total dose can be administered safely to G6PD-deficient individuals over 14 days with expected hemoglobin drops of 18 to 43% (2.7 to 6.5 g/dL drop from a baseline of 15 g/dL).

**CLINICAL TRIALS:**

This study is registered with the Thai Clinical Trials Registry (TCTR) as TCTR20170830002 and TCTR20220317004.

## INTRODUCTION

Glucose-6-phosphate dehydrogenase (G6PD) deficiency is the most common enzymopathy in humans (1). It is prevalent in areas where malaria is, or once was, endemic. G6PD deficiency is the major obstacle to the control and elimination of relapsing vivax malaria. *Plasmodium vivax* is now the predominant cause of malaria in much of Asia, the Americas, and the Horn of Africa (2). The 8-aminoquinoline drugs, primaquine and tafenoquine, are the only available antimalarial drugs which have anti-relapse activity (radical cure) in vivax malaria, but they cause dose-dependent hemolysis in G6PD deficiency (3). Because G6PD testing is often not available in routine care, patients are not prescribed 8-aminoquinoline treatment, resulting in substantial morbidity from relapses and contributing to mortality (4).

Radical curative efficacy is proportional to the total dose of primaquine administered. The World Health Organization (WHO) recommends that in patients with *P. vivax* or *P. ovale* malaria who are G6PD-deficient (usually defined as <30% of the adjusted male median enzyme activity), primaquine should be given over 8 weeks as a weekly dose of 0.75 mg base/kg (5). The rationale for this dosing regimen is based on the pharmacodynamics of 8-aminoquinoline-induced hemolysis. Mutations in the *G6PD* gene causing G6PD deficiency result in an unstable enzyme. Older red blood cells become G6PD depleted and thus more vulnerable to oxidative hemolysis (6, 7). The 0.75 mg/kg (adult dose 45 mg) weekly dosing regimen was designed to provide sufficient time for compensatory erythropoiesis which reduces the overall fall in hemoglobin, but at the expense of a protracted (8 weeks) treatment course. This 8-weekly dosing regimen was chosen on the basis of a small hematological study in three individuals who most likely had *G6PD* African A− variants (8). Its safety has not been established in patients with more severe variants of G6PD deficiency. Efficacy depends on good adherence over the 8 weeks, but nearly all the hemolytic risk is incurred from the first doses, requiring clinical monitoring (9, 10).

To develop shorter primaquine dosing regimens with acceptable safety profiles for G6PD-deficient patients with vivax malaria, we compared ascending primaquine dose regimens with a single high dose of primaquine (45 mg) in hemizygote G6PD-deficient male volunteers in Thailand (11). Ascending primaquine dose regimens are theoretically the safest way of administering radical curative dose regimens (12). By inducing “slow burn” hemolysis, they allow time for compensatory erythropoiesis, enabling the older red cell population to be replaced by younger cells with higher intraerythrocytic G6PD activities and increased oxidant resistance. In order to characterize the red cell dynamics following ascending primaquine doses, we developed a within-host Bayesian pharmacodynamic model of red blood cell production and turnover in G6PD deficiency. We fitted this model to the data from the healthy volunteer study and inferred the primaquine dose-dependent reduction in red cell lifespan. This model allows us to make generalizable predictions about the hemolytic response to other ascending dose primaquine regimens, in order to determine optimal dosing strategies for radical cure in patients with G6PD deficiency who have relapsing malaria.

## RESULTS

### Overview of study data

**Fig 1:**
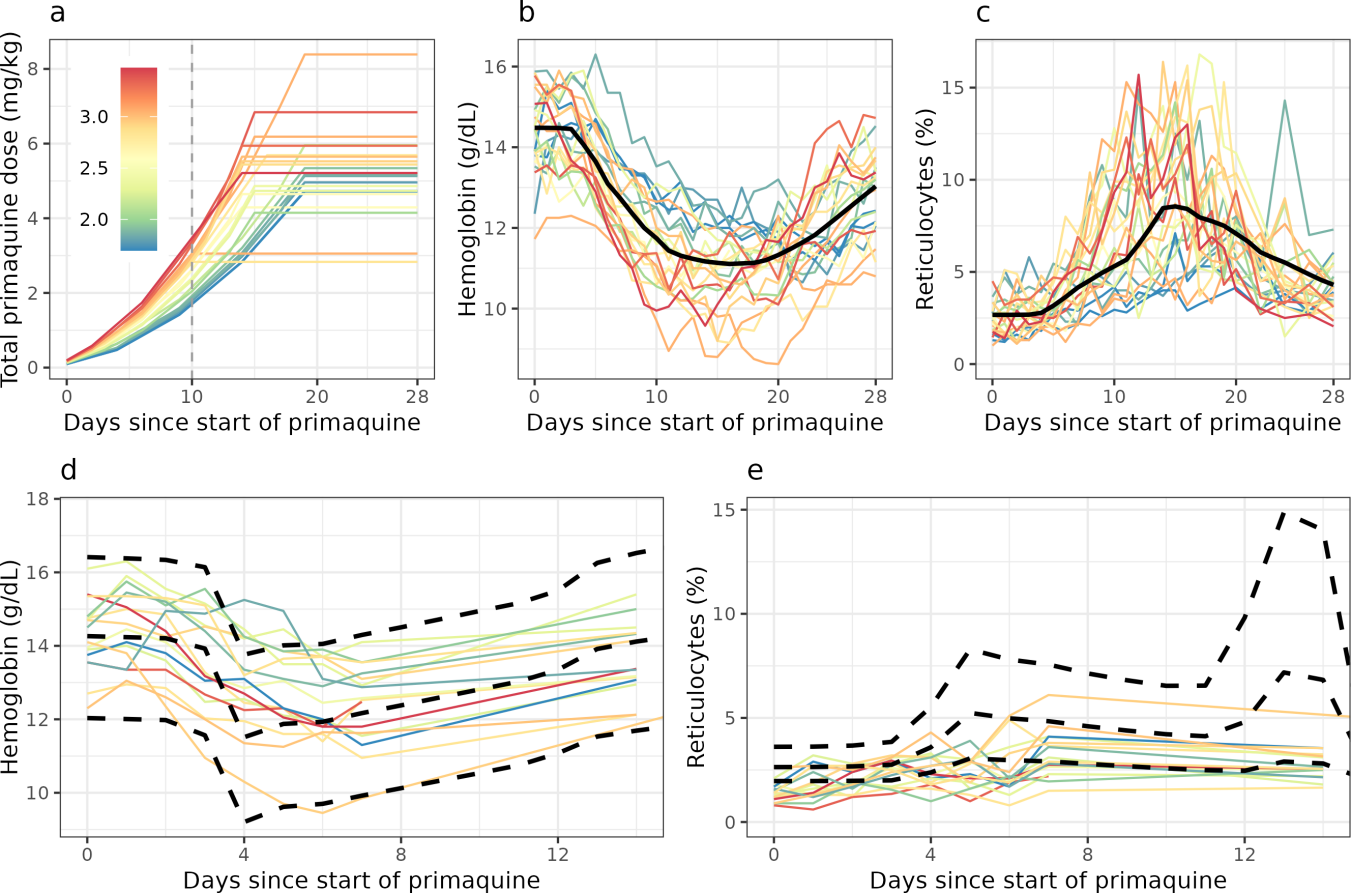
Data from the primaquine challenge study. Top panels show data from the Part 1 study (ascending dose regimens); bottom panels show the Part 2 study (single 45 mg dose). Colors correspond to the day 10 cumulative dose for the ascending dose study (proxy for how quickly the primaquine dosing was escalated), and to the mg/kg dose for the single-dose study. Solid black lines indicate the mean model fit for the ascending dose study (top panels). Dashed black lines in the bottom panels indicate the mean model predictions and 90% predictive intervals for the single-dose study, generated from the model fit for the ascending dose study.

The two studies enrolled 27 healthy male Thai and Burmese G6PD-deficient volunteers. Twenty-four volunteers received ascending primaquine doses, and 16 volunteers received single 45 mg doses, of whom 13 participated in both studies. One participant in the ascending dose study was excluded from this analysis: he only received three 7.5 mg base primaquine doses and then was withdrawn from the study due to severe back pain (prolapsed intervertebral disc—unrelated to primaquine). He was subsequently lost to follow-up. Given the very low total dose he received, his data are non-informative. The baseline characteristics of the 26 volunteers included in the analysis are shown in Table 1. Figure 1 shows the hemoglobin and reticulocyte data from the 26 volunteers by study, colored by either the day 10 cumulative dose in the ascending dose study (a proxy for how quickly the dose was increased); or the mg/kg single dose.

**TABLE 1:** Baseline characteristics of the healthy male G6PD-deficient volunteers^*a*^

	Part 1—Ascending dose	Part 2—Single 45 mg dose	Overall
*n*	23	16	26
Age (years)	32 (18–55)	34 (20–58)	32 (18–58)
Weight (kg)	64 (46–86)	64 (52–86)	64 (46–86)
G6PD genotype			
Viangchan (871G>A)	12	6	12
Mahidol (487G>A)	3	2	3
Canton (1376G>T)	4	3	5
Aures (143T>C)	1	1	1
Chinese-4 (392G>T)^*b*^	1	0	1
Orissa (131C>G)	1	1	1
Union (1360C>T)	1	2	2
Kaiping (1388G>A)	0	1	1
G6PD enzyme activity (U/g Hb)	0.15 (0–1.9)		
Hemoglobin (g/dL)	14.3 (11.8–15.8)	14.0 (12.3–15.9)	
Red cell count (×10^12^ per L)	4.9 (4.2–6.0)	5.1 (3.9–5.9)	
Reticulocyte count (%)	2.4 (1.1–4.0)	2.4 (1.0–2.9)	
CYP2D6 genotypes			
*10/*10	6	4	8
*2/*10	6	4	7
*1/*10	6	4	7
*1/*2	3	3	3
*1/*1	2	1	2

^*a*^ For the continuous variables, we show the median (range). Of the 26 volunteers included in the analysis, 13 participated in both sub-studies.

^*b*^ Also known as Quing Yan (13).

### Within-host model of primaquine-induced hemolysis

**Fig 2:**
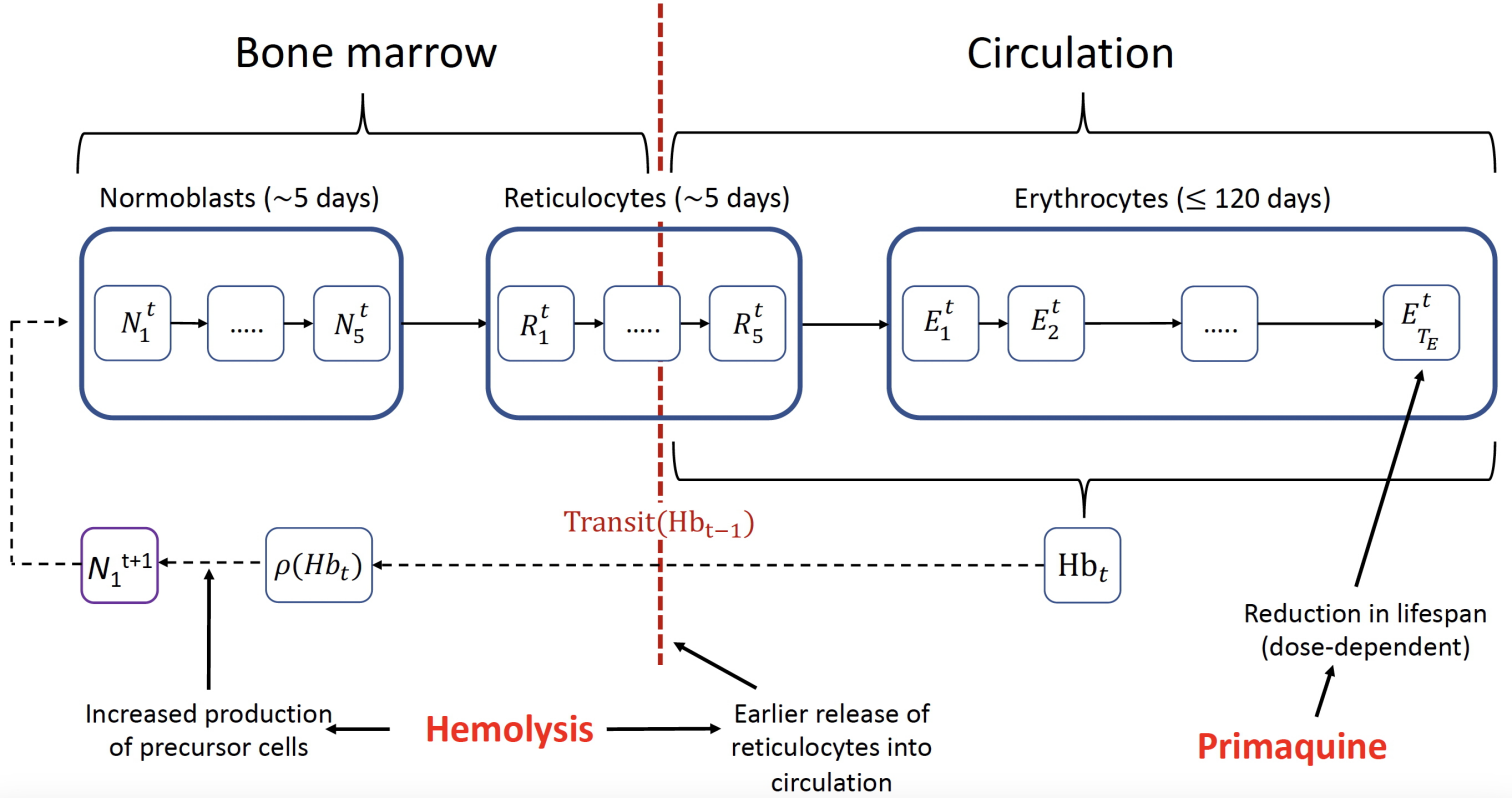
Compartmental model of red blood cell turnover and primaquine-induced hemolysis. Each compartment corresponds to 1 day and the model iterates daily. Primaquine administration reduces the lifespan of the erythrocytes resulting in hemolysis; hemolysis leads to greater production of precursor cells and earlier release of reticulocytes into circulation.

We developed a within-host model of primaquine-induced hemolysis and compensatory erythropoiesis in G6PD deficiency (see Materials and Methods for details on the model structure and implementation). Figure 2 shows an overview of the model structure. The three key assumptions driving the model behavior are: (i) the hemolytic effect of primaquine is mediated by a dose-dependent reduction in the lifespan of circulating erythrocytes; (ii) hysteresis in the hemolytic effect of primaquine can be described as a weighted sum of the doses given over the previous 10 days (non-parametric delay in effect, determined by the “effective dose,” see Materials and Methods); and (iii) compensatory erythropoiesis is a function of both the absolute difference from the steady-state hemoglobin, and the daily drop in hemoglobin (absolute hemolysis and rate of hemolysis).

**Fig 3:**
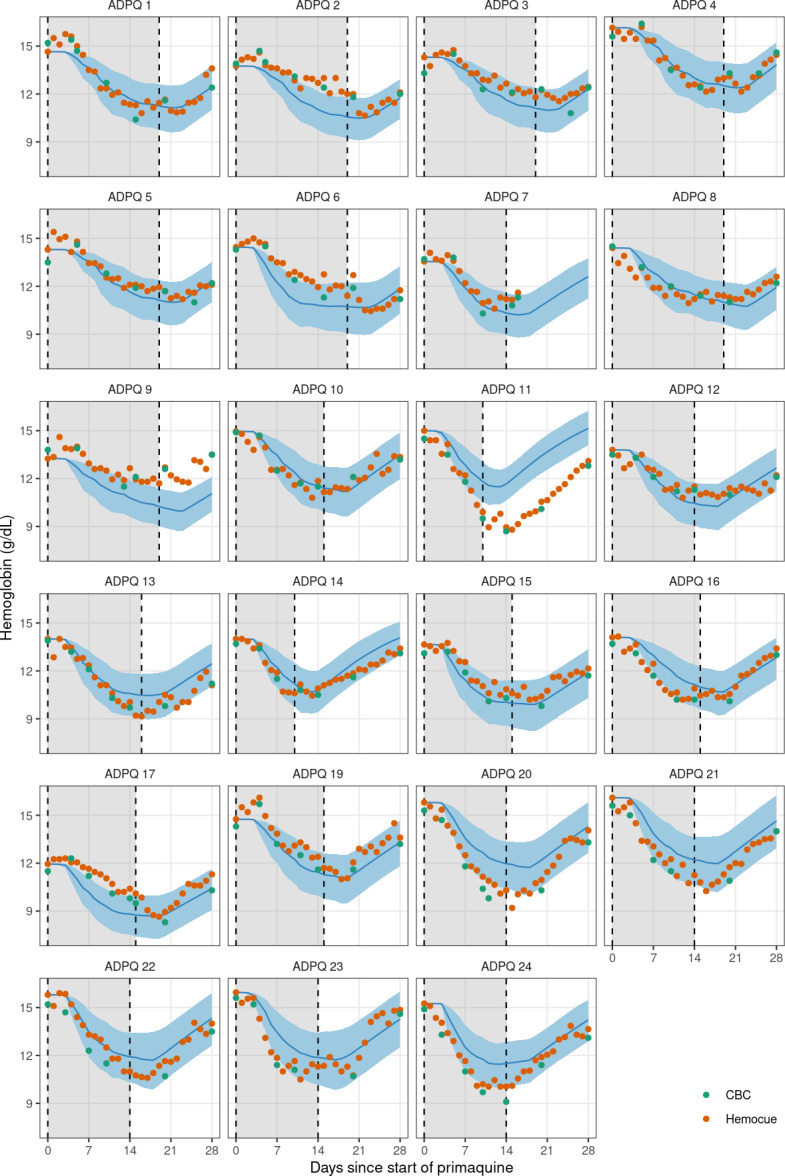
Leave-one-out predictions for the hemoglobin concentrations over time for each individual in the ascending-dose study. Dots show the source of the hemoglobin measurements (green: CBC; orange: Hemocue). Blue lines (shaded areas) show the mean model prediction (90% predictive intervals). The gray shaded area indicates the primaquine dosing period.

**Fig 4:**
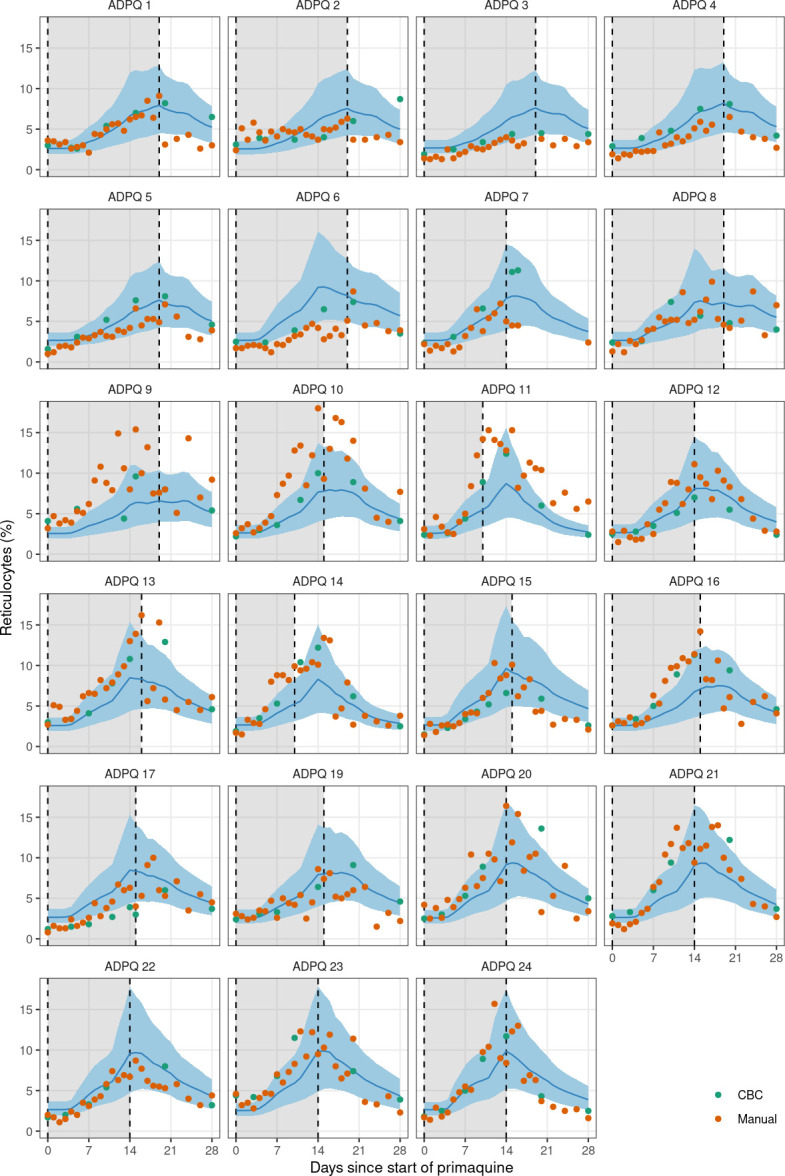
Leave-one-out predictions for the reticulocyte percentages over time for each individual in the ascending-dose study. Dots show the source of the reticulocyte measurements (green: CBC; orange: manual reading). Blue lines (shaded areas) show the mean model prediction (90% predictive intervals). The gray shaded area indicates the primaquine dosing period.

We fitted the model in a Bayesian framework to the serial hemoglobin and reticulocyte data from the 23 Thai or Burmese male volunteers who were given ascending primaquine regimens. The data included all hemoglobin and reticulocyte measurements taken between day 0 (day of first primaquine dose) and day 28, a total of 656 individual time points with 1,523 individual measurements. Estimates of key model parameters are presented in Table 2. Overall, the model captured the individual profiles for both the hemoglobin concentrations and the reticulocyte counts well. To check the predictive accuracy of the model, we re-fitted it 23 times, each time leaving one participant out, and then predicted their hemoglobin and reticulocyte profiles based on the primaquine regimen they took. This showed that the model could reliably predict out-of-sample data (Fig. 3 and 4). One individual (subject 11) had observed hemolysis which was substantially greater than the model prediction. He had the *G6PD* Union (131C>G) genotype with a normal *CYP2D6* genotype (*1/*10). Primaquine was stopped in this participant on day 10 because he met the prespecified stopping rule (40% reduction from baseline hemoglobin).

**TABLE 2:** Summary of key parameter estimates from the model fitted to the ascending dose study^*a*^

Parameter	Population mean (95% CrI)	Inter-individual variation (10th to 90th percentiles)
Steady-state hemoglobin (g/dL)	14.5 (14.1–14.9)	13.3–15.7
Steady-state RBC^*b*^ lifespan (days)	60 (55–65)	46–74
Max RBC lifespan reduction (%)	36 (31–41)	25–47
Half-maximal effect dose (mg/kg)	0.18 (0.15–0.22)	0.12–0.28
Dose-response slope	2.25 (1.81–2.69)	—^*c*^

^*a*^ We report population parameters as the posterior mean (95% credible intervals [CrIs]). Inter-individual variation is reported as 80% predictive intervals.

^*b*^ RBC, red blood cell.

^*c*^ No inter-individual variation was estimated for the dose-response slope coefficient.

The model was then used to predict expected hemoglobin reductions after giving a single 45 mg primaquine dose for the 16 individuals who participated in the second study (Fig. 1d and e). The model predicted that the nadir hemoglobin would occur on day 4, whereas the observed nadir was on day 6. The model predicted a substantial decrease in hemoglobin on day 4 (2.4 g/dL), when there was a near-maximal reduction in RBC lifespan despite the “effective dose” being only 50% of the administered 45 mg dose. This predicted daily decrease was larger than the observed decreases (Fig. 1d) and as a consequence, the model over-predicted the increase in reticulocyte production (as this is primarily driven by the rate of hemolysis). This resulted in an over-estimation of the reticulocyte percentage on day 13 (Fig. 1e). When fitting the model to both data sets jointly (ascending dose regimens and single dose), the model could not capture the exact hemoglobin and reticulocyte kinetics in both scenarios, indicating a problem with the parameterization of the hysteresis in hemolytic effect.

The model estimated that in these non-anemic healthy G6PD-deficient adult males, the major driver of increased erythropoiesis was the rate of hemoglobin decline rather than the absolute fall (absolute decrease from baseline). Peak reticulocytosis occurred approximately at the same time as the observed hemoglobin nadir in most volunteers. This could only be explained by the model when we added an explanatory covariate in the bone marrow production function: the change in hemoglobin over the previous 24 h.

Thirteen volunteers (50%) participated in both studies with over 1 year in between. In theory, these healthy participants should have had the same fundamental parameters (e.g., steady-state hemoglobin, expected red cell lifespan, and primaquine dose-response). However, several participants had considerably different baseline hemoglobin values, and markedly different responses to primaquine were observed. For example, subject 11, who was a notable outlier in terms of primaquine-induced hemolysis in the ascending dose study, had a fall of only 3.4 g/dL following the single dose (almost 3 years later), less than expected under the model. His baseline hemoglobin was nearly identical in Parts 1 and 2 (14.7 and 14.5 g/dL, respectively), but the reticulocyte count was lower in the single-dose study (2.4% and 1.4%, respectively).

### Primaquine-induced red cell age reduction

The primary aim of the within-host model was to estimate the relationship between primaquine dose (in mg/kg base equivalent) and the reduction in the circulating red cell lifespan. If this can be estimated reliably then generalizable predictions can be made regarding the effect of a specific dose regimen. The mean steady-state erythrocyte lifespan in these G6PD-deficient volunteers was estimated at approximately 60 days (95% credible interval [CrI]: 55–65), with 80% of individuals having an estimated red cell lifespan between 38 and 82 days. These estimates are considerably shorter than for G6PD normal individuals, usually quoted as approximately 120 days (14), as evidenced by the high mean reticulocyte counts at enrolment. This indicates a higher average erythrocyte turnover in healthy subjects with G6PD deficiency (11).

**Fig 5:**
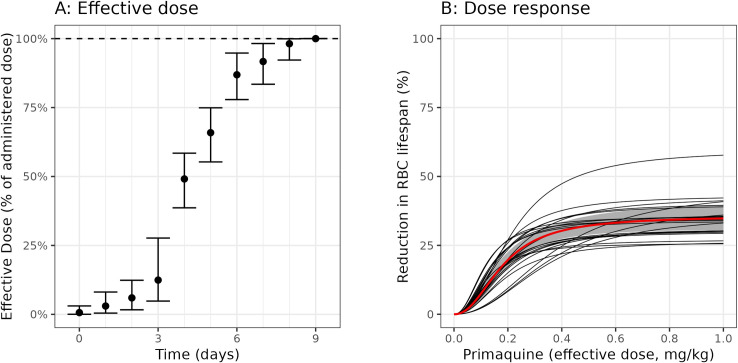
Posterior estimate of the pharmacodynamic model of primaquine-induced hemolysis. Panel A shows the delay in reaching the full effect when administering a fixed daily primaquine dose. Bars show 95% credible intervals (CrIs). Panel B shows the relationship between the effective dose and the reduction in the erythrocyte lifespan. The black lines show mean posterior dose-response curves for each of the 23 participants; the red line (gray shaded area) shows the population mean response (95% CrI). RBC, red blood cell.

Figure 5 panels A and B show the estimated delay in effect and dose-response under the model fitted to the ascending-dose regimen data. If a fixed daily dose was given, it would take approximately 1 week to reach maximal effect. Near-maximal effects are achieved with doses of 0.4 mg/kg or above (25 mg in a 60 kg adult), although there is considerable inter-individual variation both in the slope and the maximal effects estimated across the participants. Subject 11, who was a clear outlier in his hemolytic response to primaquine, had an estimated 60% reduction in circulating erythrocyte lifespan, from 52 days (95% CrI: 46–58) to 22 days (95% CrI: 17–26).

### Optimal ascending dose regimen

**Fig 6:**
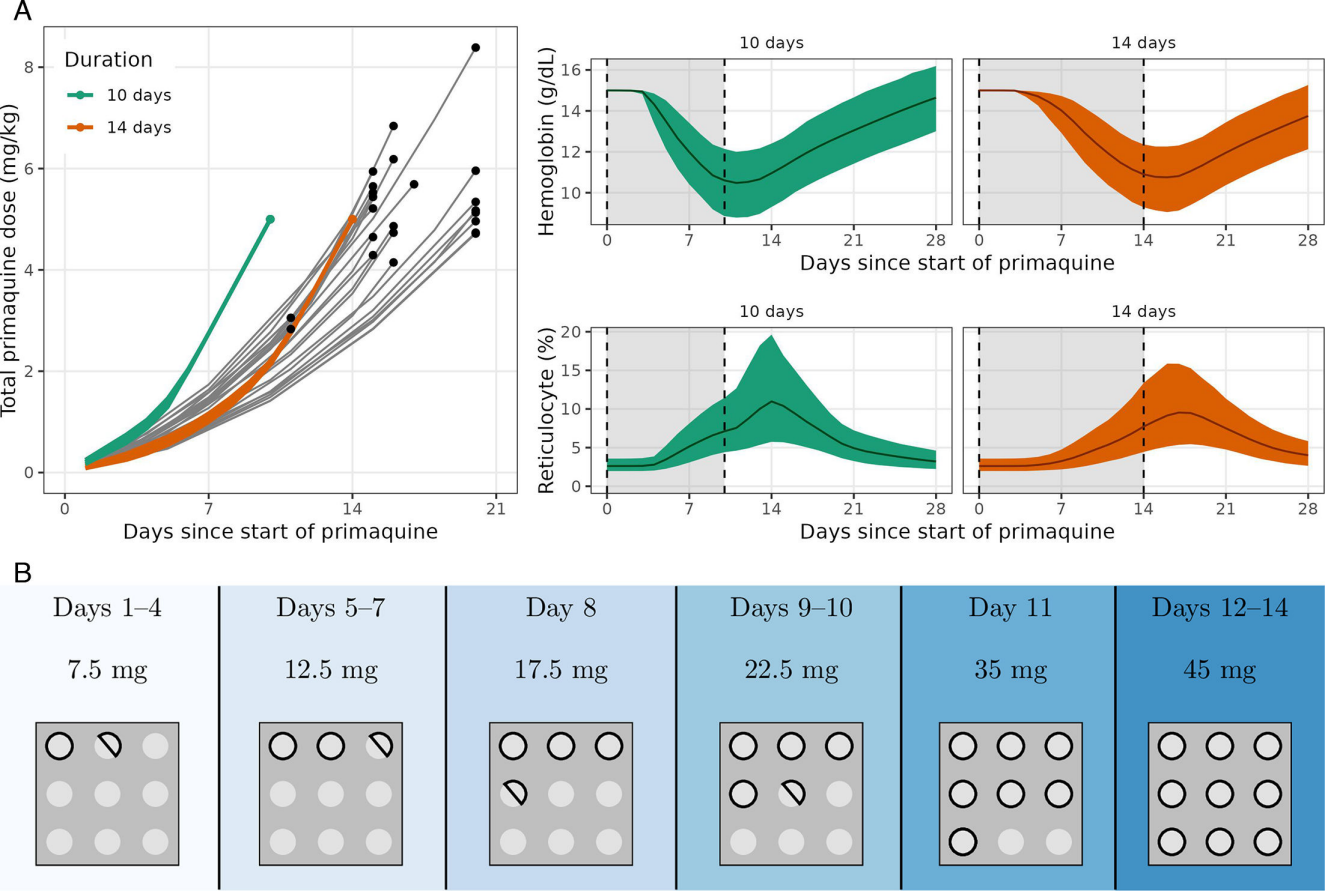
The optimal 10-day and 14-day primaquine ascending dose regimens. Panel A, left: A comparison of the optimal regimens (colored lines) to the regimens administered to each participant in the ascending dose study (gray lines). Points indicate the end of each regimen. Panel A, right: The predicted daily hemoglobin for the optimal regimens (upper panel) and the predicted daily reticulocyte levels for the optimal regimens (lower panel). Panel B: the predicted safest ascending dose regimen that comprises no more than six different dose sizes. This assumes tablet sizes of 5 mg which can be halved. This regimen is almost as safe as the optimal regimen; only 4% of individuals are predicted to have maximal daily falls over 1 g/dL (cf. 3%).

Total primaquine dose is the primary determinant of radical curative efficacy in relapsing malaria. A large individual patient data meta-analysis estimated that total doses between 5 and 7 mg/kg achieved near-maximal reductions in the relative risk of *P. vivax* recurrence over 6 months, irrespective of geographic origin and transmission intensity (15). Based on this result, we used the within-host model to estimate how a target total dose of 5 mg/kg could be administered optimally to a G6PD-deficient hemizygote male over a period of 14 days or less. We defined optimal as the regimen which minimizes the probability under the model that the individual will have a hemoglobin drop of >1 g/dL in a single day. Figure 6 shows the estimated optimal ascending dose regimens for 5 mg/kg administered over 10 or 14 days. It is not possible to administer 5 mg/kg primaquine total dose over 10 days safely. Nearly 50% of individuals are predicted to experience a maximal daily fall >1 g/dL. However, 5 mg/kg can in theory be administered safely over 14 days, allowing time for additional compensatory erythropoiesis. The estimated optimal ascending dose regimen is similar to regimens given to some participants in the healthy volunteer trial, albeit with slower increases at the start and faster increases at the end of the 2-week regimen. Under this regimen, only 3% of individuals are predicted to have maximal daily falls over 1 g/dL. For an individual with a baseline hemoglobin of 15 g/dL, the mean predicted overall drop in hemoglobin is 4.4 g/dL (95% predictive interval: 2.7–6.5 g/dL), with the nadir hemoglobin occurring between days 13 and 17.

## DISCUSSION

Our within-host model of red cell turnover and primaquine-induced hemolysis, based on detailed serial hemoglobin and reticulocyte data from the ascending primaquine dose study in healthy Thai or Burmese volunteers, suggests that it is theoretically possible to deliver effective primaquine-based radical cure in patients with moderate severity variants of G6PD deficiency within 2 weeks. This would be a substantial improvement in radical cure delivery for an important patient subgroup. In areas where G6PD deficiency is common this could affect the treatment of over 10% of individuals (16). Shorter radical cure treatment for these patients would enhance treatment adherence, reduce relapse rates, and be more practical. If a 2-week radical curative regimen was shown to be safe in G6PD-deficient vivax malaria patients, this would reduce the duration of treatment by a factor of 4 (currently, an 8-week regimen is recommended). The data and model suggest that the shorter ascending regimen would be safer than the 8-weekly regimens (45 mg per week), where all the risk is incurred with the first 45 mg dose, but no benefit is incurred until later in the course (efficacy is dependent on the total dose). The model-predicted optimal ascending dose regimen (Fig. 6) will now be tested in a Phase 2 trial in symptomatic vivax patients, with hemolytic safety as the primary outcome.

The model structure is based on a good understanding of the main underlying cellular mechanisms of oxidant hemolysis in G6PD deficiency which were elucidated from clinical studies conducted over 50 years ago (7, 17). An important result is the characterization of the substantial intra- and inter-subject variability in hemolytic response. It is well known that G6PD levels vary substantially both within and between individuals. The high basal reticulocyte counts indicate that under normal conditions G6PD-deficient subjects have increased erythrocyte turnover. Presumably, changing environmental and nutritional oxidant stresses result in variation in the proportion of the erythrocyte population which is susceptible to further (iatrogenic) oxidant hemolysis. Patients who are anemic as a result of malaria have already lost their most oxidant sensitive (i.e., older) erythrocytes (18). Thus the reductions in hemoglobin that result from primaquine oxidant hemolysis in G6PD-deficient patients with acute malaria are expected to be less than those observed in this study of healthy subjects. This is important because the main life-threatening dangers of acute hemolysis are hemoglobinuric renal failure (proportional to the fall in hemoglobin) and severe anemia (which depends on the fall, the initial hemoglobin concentration, and the chronicity of any preceding anemia). Despite the substantial intra- and inter-individual variation, a mathematical model based upon the known hemolytic pharmacodynamics fitted well to the documented changes in hemoglobin and reticulocyte counts in the ascending dose primaquine-challenged G6PD-deficient volunteers. We show that a total dose 5 mg/kg of primaquine, which is sufficient for radical cure in most patients in endemic areas (15), can be administered safely over 2 weeks to subjects with moderate severity G6PD deficiency. Only a minority of individuals are predicted to have large daily falls in hemoglobin under this regimen.

Although potentially dangerous in G6PD-deficient individuals, billions of individuals have been given primaquine over the past 70 years, with no G6PD deficiency testing in the majority (19, 20). Primaquine has been used in mass treatments in areas with a high prevalence of *G6PD* deficiency mutations (21). Primaquine-induced hemolysis is inevitable in G6PD deficiency, but it can be controlled. If iatrogenic hemolysis is balanced by erythropoiesis, the red cell population becomes progressively younger (and more resistant to oxidant stresses) with only a modest and temporary anemia. This is because hemolysis is greater in older erythrocytes. G6PD deficiency reflects an unstable enzyme that degrades more rapidly than in normal subjects. The different inherited genetic *G6PD* variants encode enzymes that vary in their stabilities or catalytic activities, affecting the proportion of circulating erythrocytes which are vulnerable to oxidant hemolysis (22). The resulting hemolysis ranges from less severe in African A− variants to more severe in the common Mediterranean variant. However, within each genotype, there is substantial phenotypic variability so serious hemolysis can occur even with the less severe variants (22). Titrating primaquine dosing against hemolysis reduces the risk of dangerous hemolytic anemia.

The choice of a radical cure dosing regimen for G6PD deficiency depends on many factors. First, the benefits of preventing relapse must outweigh the risk of hemolysis (10). The prevalence of the different *G6PD* genetic variants and their associated degree of G6PD deficiency varies substantially [1], and vivax relapse rates also vary from approximately 20% to nearly 100% (15, 23). Second, there is wide intra- and inter-individual variability in hemolytic responses, requiring a reasonably wide margin of safety. Third, complex drug regimens may result in poor adherence, and could risk unintentional overdosing. Fourth, the longer the duration of treatment, the less likely it will be completed (24). These studies show that primaquine can be given more safely to G6PD-deficient patients. While testing for G6PD deficiency is increasing, it is still unavailable outside hospitals in most vivax malaria-endemic regions. The ascending dose regimens described here could be given safely to all patients in areas where moderate severity variants are prevalent and G6PD testing is unavailable. Blister packing of the regimens with clear explanations would facilitate adherence. Field studies to assess feasibility, adherence and safety are now warranted.

A key limitation of the primaquine-induced hemolysis red blood cell model is the lack of adequate characterization of hysteresis in the pharmacodynamic effect. Primaquine is a pro-drug for which both the parent compound and the active metabolites are rapidly eliminated (half-life <12 h). However, there is a long delay between drug administration and hemolysis, as shown in the single-dose study (nadir hemoglobin occurs almost 1 week later) (11). Our model is phenomenological with respect to the delay in effect. We do not mechanistically model this delay in effect, which is driven by depletion of the erythrocyte’s G6PD mediated antioxidant defense. The non-parametric weighting of previous doses providing an “effective dose” captures the profiles observed in the ascending dose data but does not fully capture the single-dose data. An alternative modeling approach would be to parameterize enzyme decay over time, and then have hemolysis occurring as a function of red cell enzyme content. A major difficulty for this mechanistic approach is the lack of enzyme activity data at the single red cell level, and thus what the decay looks like (and how much variation there is across red cells). A simple version of this model was tried but we could not get the posterior distributions of the parameters to converge. Another limitation is the lack of understanding regarding the considerable intra- and inter-individual variability in hemolytic response. We do not have a good biomarker predictive of this variability. Intra- and inter-individual variability may be driven by differences in recent hemolytic insults, for example, from diet or infection. Our model assumes that at enrolment the distribution of circulating red cell ages is approximately uniform. Deviations from this assumption have important consequences on the impact of primaquine administration. Most importantly, this limits the ability to predict primaquine-induced hemolysis reliably in patients with vivax malaria. Malaria causes hemolysis of non-parasitized older red cells (which are removed by the spleen) which would be expected to attenuate the effect of primaquine (18). However, in some patients with hematinic deficiencies, there may be an attenuated bone marrow response. Extrapolating from healthy volunteers to malaria patients is uncertain and data from patients are now needed to calibrate the model.

This model allows the prediction of the hemolytic dose-response relationship in subjects with Southeast Asian variants of G6PD deficiency. This should be applicable to other variants of similar severity and will overestimate hemolysis in milder variants. It cannot be extrapolated directly to more severe variants (e.g., *G6PD* Mediterranean). However, the large variation of phenotypes within genotypes means that even individuals with less severe variants may occasionally experience severe hemolysis. More information is needed to calibrate these risks. The proposed ascending dose regimen should now be tested for safety in patients with *P. vivax* malaria.

## MATERIALS AND METHODS

### Clinical study

The Primaquine Challenge study was a two-part regimen-adaptive open-label study that aimed to assess the safety and tolerability of ascending primaquine dose regimens in healthy Thai and Burmese male G6PD-deficient volunteers. Clinical details have been published previously (11). All volunteers provided fully informed written consent and agreed to all study procedures. The two parts of this study were approved as separate studies. Both parts were approved by the Faculty of Tropical Medicine’s Ethics Committee (MUTM 2017-036-01 and MUTM 2021-031-02) and the Oxford Tropical Research Ethics Committee (OxTREC, number 48-16). The study protocols were pre-registered on the Thai Clinical Trial Registry (TCTR, numbers TCTR20170830002 and TCTR20220317004).

In brief, the study subjects were healthy male adult volunteers (18–65 years of age) recruited in Bangkok (Faculty of Tropical Medicine, Mahidol University) with G6PD deficiency confirmed by a validated quantitative spectrophotometric G6PD assay (enzyme activity <30%), and with a known genotype. Individuals with the G6PD Mediterranean variant (C563T) or any previously uncharacterized mutations were excluded from the study. A total of 27 individuals were recruited over 4 years (the study was interrupted substantially by the COVID-19 pandemic). The study had two parts. In Part 1, ascending primaquine doses over 15–20 days were given (daily doses ranging between 7.5 and 45 mg base). In Part 2, single 45 mg doses were given. Twenty-four individuals participated in Part 1. COVID-19 mitigation measures interrupted the end of Part 1 and considerably delayed finishing the study. Thirteen subjects from Part 1 also participated in Part 2, and three additional subjects were enrolled to capture inter-individual variability better following the single 45 mg dose.

All subjects were admitted to the Phase 1 unit at the Hospital of Tropical Diseases during the period of drug administration (between 15 and 20 days) for Part 1, and for 1 week following the single dose for Part 2. All primaquine doses were directly observed. In Part 1, follow-up occurred over 49 days following the first primaquine dose; and in Part 2 over 14 days. Venous blood samples for complete blood counts (CBCs) were taken at screening and then every 4–5 days during drug administration. Finger prick blood samples were taken daily during drug administration and at follow-up visits for manual reticulocyte count readings and hemocue hemoglobin measurement. The primaquine regimens administered were adapted throughout the trial based on accrued data, perceived safety by the study investigators and using a set of pre-specified rules (11). The aim of Part 1 was to determine a regimen that resulted in a steady decrease in hemoglobin during primaquine dosing with a total decrease that was less than 40% of baseline; this hemoglobin drop was then compared with that resulting from a single high dose (45 mg base equivalent) primaquine (Part 2).

### Compartmental model of red blood cell dynamics

Red blood cells are produced through a highly regulated process (erythropoiesis), which starts with the commitment of hemopoietic precursors and the generation of immature nucleated red cells (normoblasts) in the bone marrow. These normoblasts lose their nuclei and mature into reticulocytes, which are then released into the bloodstream. These cells lose their “reticulated” appearance (resulting from residual mRNA) after 1–2 days and become mature erythrocytes. At steady state in normal adults, reticulocytes constitute approximately 1% of all circulating red blood cells. Loss of red blood cells (in this case from hemolysis) triggers a feedback response resulting in increased production in the bone marrow and an earlier release of reticulocytes into circulation (17). An increased reticulocyte count can thus reflect either hemolysis that has just occurred (i.e., reticulocytes are a greater proportion of all circulating red blood cells), a recently increased bone marrow production, or both (17). Reticulocyte counts are thus an important proxy marker of bone marrow erythropoietic activity.

In this work, we update a previously published model of red blood cell dynamics (12) to infer the dose-dependent hemolytic effect of primaquine in G6PD deficiency, and characterize the dynamics of red blood cell production and response to hemolysis. The key underlying model assumption is that primaquine-induced hemolysis is red cell age-dependent, that is, the older cells hemolyze first. This is parameterized in the model as a dose-dependent reduction in the red blood cell lifespan.

### Steady-state dynamics

We denote values at steady-state with the superscript ${}^{*}$, for example, the steady-state hemoglobin concentration is denoted ${\text{Hb}}^{*}$. The model captures variation over time in hemoglobin concentrations and in the proportion of circulating red cells which are reticulocytes, indexed by t. We assume that at the first timepoint t=0 (i.e., before drug administration) the subject is at steady-state, ${\text{Hb}}_{t=0}={\text{Hb}}^{*}$. Steady-state means that over 1 day the number of red cells which are removed from the circulation equals the number liberated (and thus newly produced) by the bone marrow. The model is parameterized in terms of the number of circulating red blood cells (denoted C${}_{t}$), assuming a constant one-to-one relationship between the number of circulating red blood cells and the observed hemoglobin concentration. This assumes no changes in mean corpuscular hemoglobin content. At t=0, ${\text{Hb}}_{t}=\Lambda {\text{C}}_{t}$. We note that the dynamics of the system are invariant to the value of the scaling factor Λ so we use a computationally convenient value.

The numbers of red blood cells at the different stages are modelled using non-linear difference equations broken into daily blocks. The number of red blood cells in the *i*th block of each of the three stages of red blood cell maturation at time t is denoted by $N_{i}^{t}$, $i=1,\ldots ,T_{N}$ for normoblasts; $R_{i}^{t},i=1,\ldots ,T_{R}$ for reticulocytes; and $E_{i}^{t},i=1,\ldots ,T_{E}$ for erythrocytes. $T_{N}$, $T_{R}$, and $T_{E}$ are the maximal survival ages in units of days for normoblasts, reticulocytes, and erythrocytes, respectively. For example, $N_{i}^{t}$ corresponds to the number of normoblasts that are i days old in the system at time t. Normoblasts are contained within the bone marrow (we assume no liberation). Reticulocytes are formed in the bone marrow and then emerge into circulation at a varying age, $\text{Transit}({\text{Hb}}_{t-1})$, which we model as a function of the hemoglobin concentration at the previous time point (17). At steady state in a normal individual, reticulocytes emerge from the bone marrow after approximately three and a half days (i.e., $\text{Transit}[{\text{Hb}}^{*}]=3.5$ days) and spend approximately one and a half days in circulation before becoming mature red blood cells (erythrocytes). An overview of the model structure is shown in Fig. 2.

### Release time of reticulocytes

At time t, the total number of circulating cells is given by:


$$\begin{array}{c} C_{t}=\sum_{i=\text{Transit}({\text{Hb}}_{t-1})}^{T_{R}}R_{i}^{t}+\sum_{i=1}^{T_{E}}E_{i}^{t}, \end{array}$$

The quantity $\sum_{i=\text{Transit}({\text{Hb}}_{t-1})}^{T_{R}}R_{i}^{t}$ is the total number of circulating reticulocytes. The proportion of reticulocytes in the circulation is then simply $\sum_{i=\text{Transit}({\text{Hb}}_{t-1})}^{T_{R}}R_{i}^{t}/C_{t}$. Note that this assumes Transit(⋅) takes integer values only; in the more general case, we take the fractional part of the first term in the sum.

The transit time of reticulocytes into circulation is parameterized as a simple exponential function:


$$\text{Transit}({\text{Hb}}_{t-1})=\left\{ \begin{array}{lll} 3.5e^{-k({\text{Hb}}^{*}-{\text{Hb}}_{t-1})} & \;\text{if }\;{\text{Hb}}_{t-1}<{\text{Hb}}^{*} & \\ 3.5 & \;\text{otherwise} \end{array}\right.$$

where k is the slope coefficient which determines how quickly the transit time reduces as the hemoglobin falls below steady-state; 3.5 is the steady-state value (days).

### Production of normoblasts

Hemolysis triggers an increase in the production of red blood cells in the bone marrow. This is captured by the “production function” ρ (expressed as a fold change relative to steady state). We parameterize ρ as a function of both the absolute difference in hemoglobin from steady-state (i.e., total loss of red blood cells relative to steady-state) and the change in hemoglobin compared to the previous day (reflecting the rate of hemolysis). For both components, we use second-degree polynomials:


$$\begin{array}{c} \rho ({\text{Hb}}_{t-1},{\text{Hb}}_{t-2})=1+f_{1}({\text{Hb}}^{*}-{\text{Hb}}_{t-1})+f_{2}({\text{Hb}}_{t-2}-{\text{Hb}}_{t-1}) \end{array}$$

where for s=1,2:


$$f_{s}(x)=\left\{ \begin{array}{cc} \delta_{s}^{1}x+\delta_{s}^{2}x^{2} & \;\text{if }x>0\\ 0 & \;\text{otherwise} \end{array}\right.$$

### Forward time simulation

The model iterates daily the production and release time processes to determine changes over time in hemoglobin concentration and reticulocyte counts for a given set of parameters. At time t=0, the model is initialized at steady state:


$$\begin{array}{rlr} {\text{Hb}}_{t=0}= & {\text{Hb}}^{*} & \\ N_{i}^{t=0}= & R_{j}^{t=0}=\rho^{*}\; & \text{for all }i=1,...T_{N},j=1,...,T_{R}\\ E_{i}^{t=0}= & \rho^{*}\prod_{j=1}^{i}\left(1-\Phi \left[\frac{j-T_{E}^{*}}{\sigma }\right]\right), & \text{where }i=1,...T_{E} \end{array}$$

In each subsequent time step, $\left\{N_{i}^{t},R_{i}^{t},E_{i}^{t}\right\}$ are updated from $\left\{N_{i}^{t-1},R_{i}^{t-1},E_{i}^{t-1}\right\}$ using the following difference equations:


$$\begin{array}{rlr} N_{1}^{t}= & \rho^{*}\rho (Hb_{t-1},Hb_{t-2}) & \\ R_{1}^{t}= & N_{T_{N}}^{t-1} & \\ E_{1}^{t}= & R_{T_{R}}^{t-1} & \\ N_{i}^{t}= & N_{i-1}^{t-1} & 1<i\leq T_{N}\\ R_{i}^{t}= & R_{i-1}^{t-1} & 1<i\leq T_{R}\\ E_{i}^{t}= & \left[1-\Phi \left(\frac{i-T_{E}^{*}}{\sigma }\right)\right]\times E_{i-1}^{t-1} & 1<i\leq T_{E} \end{array}$$

The value $\rho^{*}$ is an arbitrary scalar which determines the relationship between the total number of circulating red blood cells and the units of hemoglobin (i.e., determines the value of Λ); Φ is the cumulative standard normal distribution (probit function); $T_{E}^{*}\leq T_{E}$ is the expected red blood cell lifespan for the individual. The function 1−Φ parameterizes the steady-state age-dependent removal of older red blood cells as a sigmoid process. The parameter σ determines the “steepness” of this removal process when the age approaches $T_{E}^{*}$. The probit curve results in negligible decay until 3–4 standard deviations (σ) from the mean value, which in absence of drug is $T_{E}^{*}$. Thus at steady the number of erythrocytes is approximately uniformly distributed over the interval $\left[0,T_{E}^{*}\right]$.

### Primaquine-induced hemolysis in G6PD deficiency

Primaquine administration results in dose-dependent hemolysis in G6PD-deficient individuals. This represents the loss of older red blood cells which contain lower G6PD enzymatic activity, and are therefore susceptible to primaquine-induced oxidative stress (7). Delay between starting primaquine and the onset of hemolysis is attributed to depletion of the remaining intraerthrocytic anti-oxidant defences in the oldest erythrocytes. Primaquine is not the active moiety–it is converted to bioactive intermediates which cause oxidative hemolysis and the beneficial antimalarial effect. This is relatively rapid (although the degree of conversion varies between individuals), so for the purposes of this model, the primaquine dose is considered a surrogate for the amount of oxidative intermediates produced. Our model assumes oral daily primaquine administration. When modeling the time interval $\left[0,T_{\max}\right]$, we represent the primaquine regimen as a vector $\{d_{t}{\}}_{t=0}^{T_{\max}-1}$, where $d_{t}$ is the mg/kg dose administered on day t. Primaquine is metabolised rapidly and so can be modelled as daily “pulses.” However, a complicating factor is the delay between the primaquine dose administration and the resulting hemolysis (hysteresis).

#### Effective dose: delay in effect

We model hysteresis in the hemolytic effect of primaquine by defining an “effective dose” $x_{t}$ at time t which is a function of the previously administered doses. The effective dose $x_{t}$ is a time-weighted average of the previously administered doses:


$$x_{t}=\sum_{ı=0}^{D}\omega_{ı}\cdot d_{t-l}$$

We used a symmetric Dirichlet prior distribution for the weights $w=\left(w_{0},w_{1},\ldots ,w_{D}\right)$, with concentration parameter 1.

#### Dose-dependent reduction of red blood cell lifespan

We model the dose-dependent hemolytic effect of primaquine by defining the reduced erythrocyte lifespan at time t to be $T_{E}^{t}(x_{t-1})$, a function of the “effective dose” $x_{t-1}$ from the previous day. Because the effective dose is a weighted average of previous doses, this takes into account the delay in the effect. The dose-dependent effect of primaquine is modelled as an E${}_{\max}$ function of the effective dose x:


$$\begin{array}{c} {\text{E}}_{\max}(x)=\frac{\alpha \cdot x^{h}}{\beta_{50}^{h}+x^{h}} \end{array}$$

where α∈[0,1] is the maximum achievable primaquine-induced decrease in relative lifespan. The parameter $\beta_{50}$ is the half-maximal effect dose, that is, ${\text{E}}_{\max}(\beta_{50})=\alpha /2$. The parameter h is the “Hill” coefficient which determines the slope of the dose-response curve.

The reduced red blood cell lifespan at time t is thus:


$$T_{E}^{t}(x_{t-1})=T_{E}^{*}\cdot (1-{\text{E}}_{\max}[x_{t-1}])$$

Therefore, at each time step the number of erythrocytes is updated with the following difference equation:


$$E_{i}^{t}=\left[1-\Phi \left(\frac{i-T_{E}^{t}\left(x_{t-1}\right)}{\sigma }\right)\right]\cdot E_{i-1}^{t-1},\;\;\;\;\;\;1<i\leq T_{E},$$

### Optimal ascending dose regimen

An ideal primaquine regimen for individuals with G6PD deficiency would provide a radical curative dose, over a reasonable period (2 weeks as for the standard regimen), whereby falls in hemoglobin are gradual (12). A review of the available evidence suggested that a total dose of 5 mg base/kg provides near-maximal anti-relapse (radical cure) activity (15). We defined “optimal” as the regimen which minimzes the probability of a fall >1 g/dL over the course of a single day.

We used the posterior distribution over the model parameters when fitted to the ascending dose study data to identify optimal 10-day and 14-day regimens, subject to the following criteria:

- Each daily dose was a multiple of 2.5 mg (equivalent to half of a 5 mg tablet);
- Each daily dose was at least as large as the previous dose (i.e., ascending); and
- The total dose was 300 mg (equivalent to 5 mg/kg for a 60 kg individual).

There were 78,796 possible 10-day regimens, and 3,648,057 possible 14-day regimens that satisfy these criteria. We used a brute-force approach, and evaluated each candidate regimen by simulating the model forward from time t=0 for 1,000 virtual individuals. We drew 1,000 posterior samples for the population model parameters and participant-specific model parameters (individual random effects). Each individual was assigned a random body weight which was normally distributed with mean 60 and standard deviation 5. For each individual, we recorded the maximum daily drop in hemoglobin, and scored each candidate regimen by the proportion of individuals who experienced a drop of >1 g/dL.

### Model fitting

Model parameters were estimated under a Bayesian hierarchical model framework allowing for inter-individual variation in the hemolytic effect of the drug, the steady-state red blood cell lifespan, the steady-state hemoglobin, bone marrow response to hemolysis, and reticulocyte response to hemolysis (transit time from bone marrow to circulation).

### Data notation

In all the following the subscript t denotes the time in days, and i denotes the subject number. The model is fitted to four types of data: (i) CBC hemoglobin values, denoted $Y_{i,t}^{1}$; (ii) Hemocue hemoglobin values, denoted $Y_{i,t}^{2}$; (iii) CBC reticulocyte values, denoted $Z_{i,t}^{1}$; and (iv) manually read reticulocyte values, denoted $Z_{i,t}^{2}$.

The residual errors for the modelled hemoglobin concentrations were assumed to be normal; for the reticulocyte counts, they were assumed to be log-normal.


$$\begin{array}{ll} Y_{i,t}^{j}∼Normal\left[M_{Hb}\left(\theta_{i};t\right)+1\left(j=1\right)\delta_{CBC},\sigma_{Hb}^{j}\right],\;\;\;\;\;j=1,2 & \\ log\left(Z_{i,t}^{j}\right)∼Normal\left[log\left(M_{Retic}\left(\theta_{i};t\right)\right),\sigma_{Retic}\right],\;\;\;\;\;\;\;\;j=1,2 \end{array}$$

The parameter $\delta_{\text{CBC}}$ adjusts for the systematic differences in the hemoglobin measurement between CBC and Hemocue (11). $M_{\text{Hb}}(\theta_{i};t)$ and $M_{\text{Retic}}(\theta_{i};t)$ correspond to the model-predicted hemoglobin concentration and reticulocyte counts for a given individual-specific parameter vector $\theta_{i}=\theta +\eta_{i}$; θ is the set of population-level parameters:


$$\theta =\left\{T_{E}^{*},{\text{Hb}}^{*},\delta_{\text{CBC}},{\left(\sigma_{\text{Hb}}^{j}\right)}_{j=1,2},\sigma_{\text{Retic}},k,w,\alpha ,\beta_{50},h,{\left(\delta_{s}^{k}\right)}_{s=1,2}^{k=1,2}\right\}$$

$\eta_{i}$ are the individual random effects that capture variation between individuals. $\eta_{i}$ has dimension 8. Individual random effects are added to all parameters except for $\left\{\delta_{\text{CBC}},{\left(\sigma_{\text{Hb}}^{j}\right)}_{j=1,2},\sigma_{\text{Retic}},w,k,h\right\}$. The prior distribution for the individual-specific parameters was assumed to be multivariate normal with mean zero and covariance matrix Ω. Ω was decomposed into a vector of standard deviation parameters and a correlation matrix with a Cholesky prior distribution with shape parameter equal to 2 (25, 26). The prior distributions for all parameters, as well as all fixed model parameters, are provided in Table S1 in the supplemental material along with their interpretation.

Analysis was conducted in R 4.1.3 (27) and model fitting was performed using Stan 2.34 (25) and the CmdStanR package 0.7.1 (28). Four chains were implemented and 1,000 posterior samples were retained from each chain after a burn-in of 1,000 iterations, resulting in 4,000 samples per parameter for the calculation of posterior summaries. The posterior summaries calculated for each parameter were the mean of these samples and the 90% credible interval, which was calculated from the 5th and 95th percentiles of samples. About 80% of predictive intervals to quantify inter-individual variation in Table 2 were derived from the 10th and 90th percentiles of the draws for the individual-specific parameters.

Convergence was checked from trace plots and standard MCMC diagnostic criteria including the $\hat{R}$ statistic (<1.05 was considered to demonstrate acceptable convergence), and the effective sample sizes (ESS). Prior and posterior distributions were compared visually for the main parameters (supplemental Figure S2).

A total of 1,000 random samples were drawn from population red blood cell model parameters (θ) derived from the posteriors to compare different dosing regimens. Every one of the red blood cell vector of parameters corresponds to a single red blood cell profile data set, where each of these vectors and a dosing regimen of primaquine, were input into the red blood cell model in order to simulate 1,000 hemoglobin concentration and reticulocyte counts. We draw 1,000 random sets of participants’ specific red blood cell model parameters to simulate 1,000 hypothetical red blood cell profiles (hemoglobin concentration and reticulocyte counts) and to compare alternative dosing regimens of primaquine to the current regimen administered by each individual. The process outlined above was repeated for each dosing regimen.

## Data Availability

All data and code areavailable on GitHub: https://github.com/jwatowatson/Primaquine-Challenge.

## SUPPLEMENTAL MATERIAL

Supplemental material aac.01549-24-s0001.pdf Figures S1 to S4.

ASM does not own the copyrights to Supplemental Material that may be linked to, or accessed through, an article. The authors have granted ASM a non-exclusive, world-wide license to publish the Supplemental Material files. Please contact the corresponding author directly for reuse.
