# Supplementary material for: Within-host modeling of primaquine-induced hemolysis in hemizygote glucose-6-phosphate dehydrogenase deficient healthy volunteers
Source: Antimicrob Agents Chemother. 2025 Feb 24;69(4):e01549-24. doi: 10.1128/aac.01549-24 (PMC11963541; doi:10.1128/aac.01549-24)
Supplement: Supplemental material — Figures S1 to S4. [file aac.01549-24-s0001.pdf]

Supplementary Materials: within-host modelling of primaquine-induced haemolysis in hemizygote glucose-6-phosphate dehydrogenase deficient healthy volunteers

James A Watson et al.

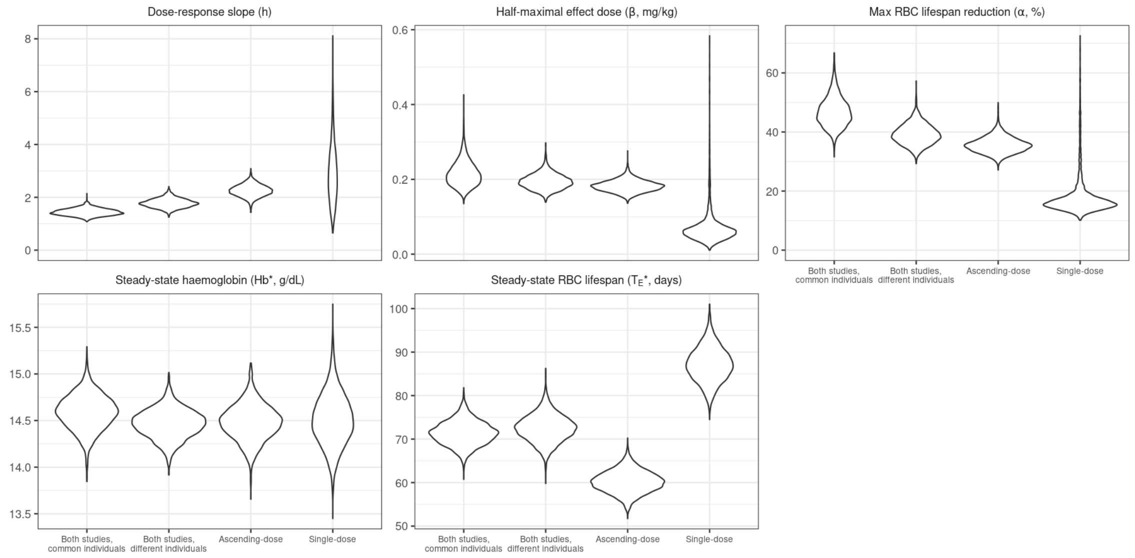

Figure S1: Comparison of the marginal posterior distributions for key model parameters when fitting to (a) both studies, assuming that individuals enrolled in both have identical parameters; (b) both studies, assuming that individuals enrolled in both have independent parameters; (c) only the ascending dose study; and (d) only the single dose study. There are clear differences between the dose responses and RBC lifespans estimated from the single dose study and from the ascending dose study. In particular, the single dose study estimates a much longer steady-state RBC lifespan, and a much smaller dose response that is triggered by very low doses.

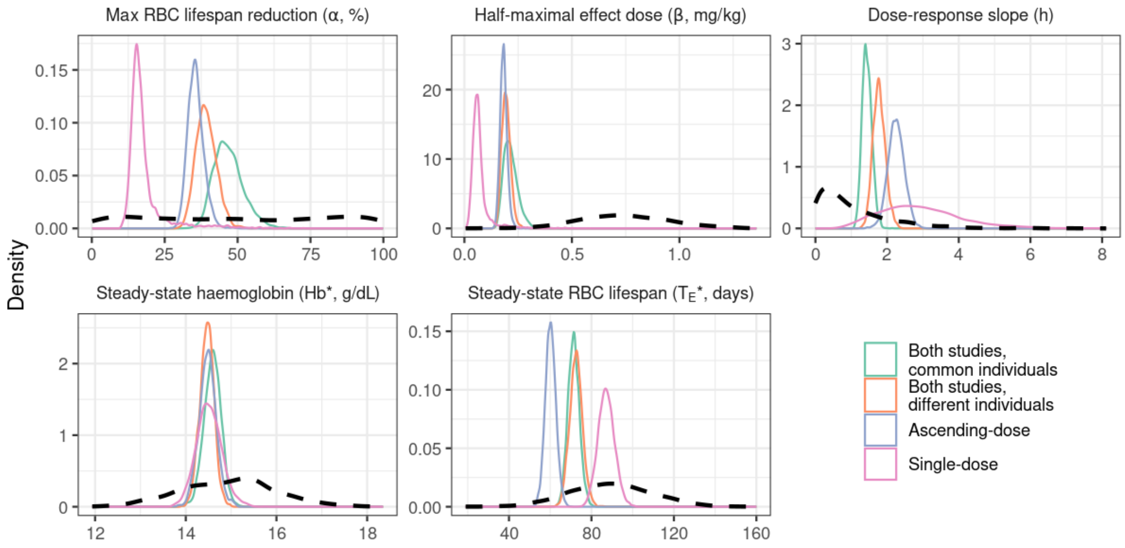

Figure S2: Comparison of the marginal posterior distributions for key model parameters to the prior distributions. Marginal posterior distributions are shown when fitting to (a) both studies, assuming that individuals enrolled in both have identical parameters; (b) both studies, assuming that individuals enrolled in both have independent parameters; (c) only the ascending dose study; and (d) only the single dose study.

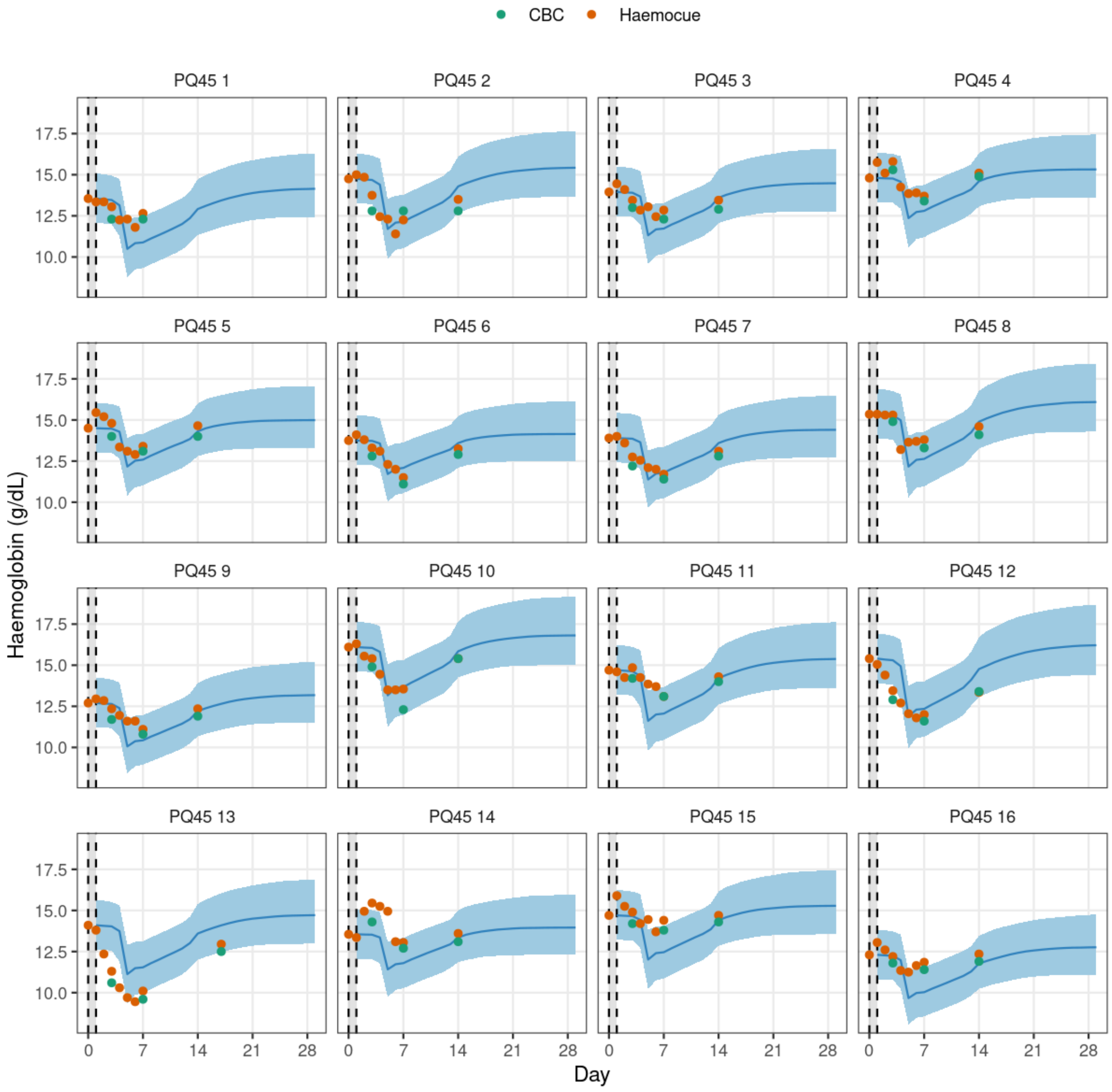

Figure S3: Haemoglobin predictions following the single 45mg dose, under the model fitted to ascending-dose study. The model predicts a rapid drop in Hb several days after the single dose; this is consistent with the data from some individuals (e.g., PQ45 2 and PQ45 10), but other individuals experienced a smaller, more gradual decrease in Hb (e.g., PQ45 1 and PQ45 11), an earlier drop in Hb (e.g., PQ45 12 and PQ45 13), or even no decrease in Hb (e.g., PQ45 14 and PQ45 15).

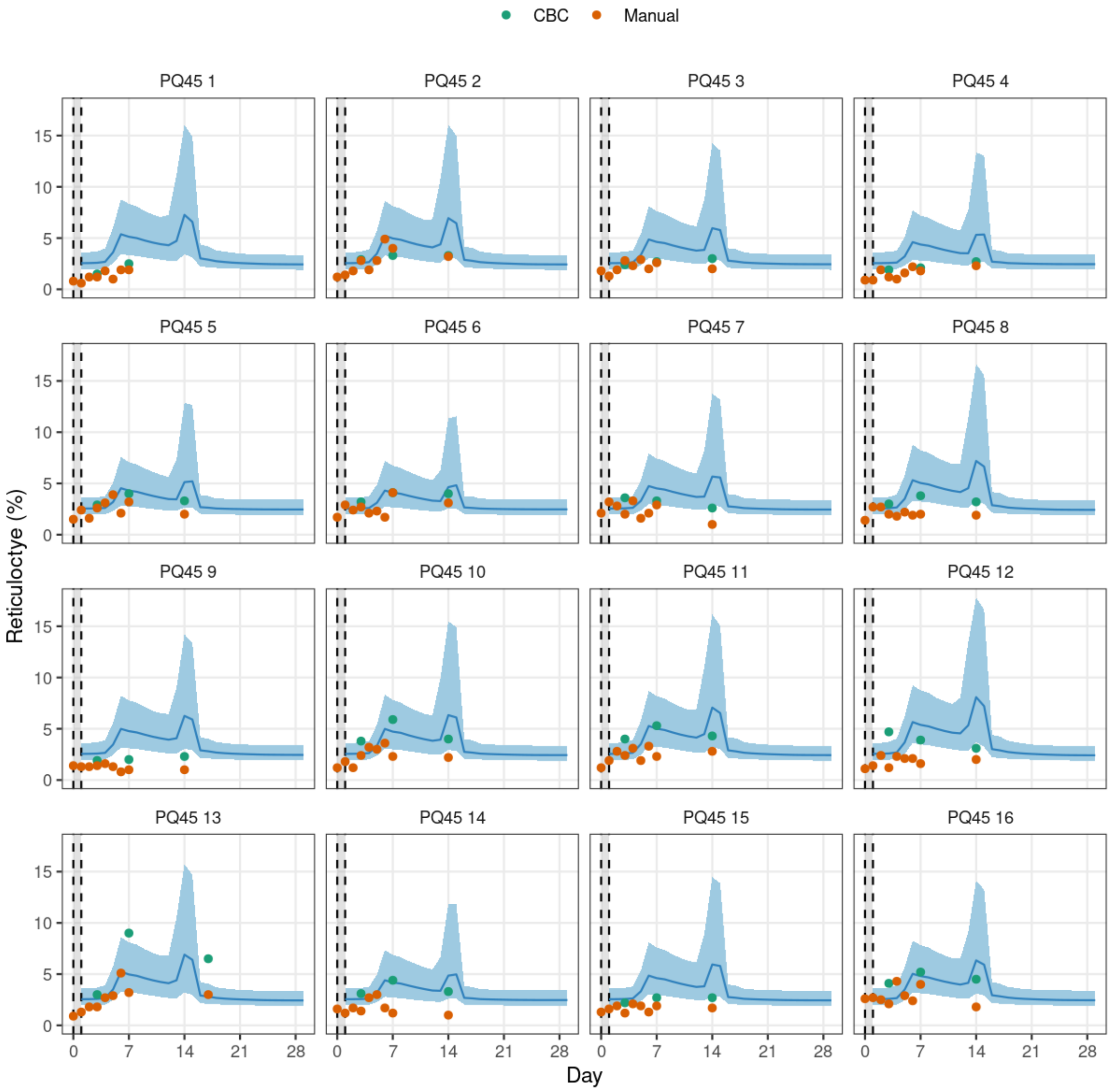

Figure S4: Reticulocyte predictions following the single 45mg dose, under the model fitted to ascending-dose study. The predictions are in reasonable agreement with the measurements taken over days 0 to 7, but the measurements taken on day 14 (day 17 for individual PQ45 13) do not provide evidence of a short, rapid increase in reticulocytes.

Parameter	Unit	Description	Population parameter prior	Prior on variance of random effect
T_E^*	days	steady state red cell lifespan	$T_E^* \sim \text{Normal}(90, 20)$	Exponential(1)
Hb^*	g/dL	steady state haemoglobin	$\text{Hb}^* \sim \text{Normal}(15, 1)$	$\text{Normal}(1, 1)$
δ_{CBC}	g/dL	Systematic difference in CBC versus Haemocue haemoglobin measurements	$\delta_{\text{CBC}} \sim \text{Normal}(0, 1)$	-
k	unitless	slope parameter for reticulocyte transit function	$\log(k) \sim \text{Normal}(-1, 2)$	-
δ_s^k	unitless	change in normoblast production as a function of changes in haemoglobin	$\delta_s^k \sim \text{Exponential}(10)$	Exponential(10)
Transit^*	days	number of days reticulocytes stay in bone marrow at steady state	fixed: 3.5	-
h	unitless	slope parameter of the dose-response curve	$h \sim \text{Exponential}(1)$	-
$\mathbf{w}$	unitless (sum to 1)	weights to construct delay in effect	$\mathbf{w} \sim \text{Dirichlet}(\mathbf{1})$	-
α	proportion	maximum relative decrease in the red cell lifespan	$\text{InvLogit}(\alpha) \sim \text{Normal}(0, 2)$	$\text{Normal}(0.5, 0.5)$
β_{50}	mg/kg	Primaquine dose resulting in half-maximal decrease in red cell lifespan	$\beta_{50} \sim \text{Normal}(0.75, 0.2)$	Exponential(10)
$\left(\sigma_{\text{Hb}}^j\right)_{j=1,2}$	g/dL	Standard deviation of CBC (j=1) and Haemocue (j=2) haemoglobin measurement error	$\sigma_{\text{Hb}}^j \sim \text{Exponential}(1)$	-
σ_{Retic}	log proportion	Standard deviation of the reticulocyte measurement error (proportional error, log scale)	$\sigma_{\text{Retic}} \sim \text{Normal}(0.5, 0.5)$	-
σ	days	nuisance parameter determining death rate of older red cells	fixed: 3	-
T_N	days	Normoblast lifespan	fixed: 5	-
T_R	days	Reticulocyte lifespan	fixed: 5	-
T_E	days	Maximal erythrocyte lifespan	fixed: 140	-

Table S1: Prior distributions for model.
